# Knowledge, Attitudes, and Practices Toward COVID-19 Among Chinese Teachers, Shenzhen: An Online Cross-sectional Study During the Global Outbreak of COVID-19

**DOI:** 10.3389/fpubh.2021.706830

**Published:** 2021-08-20

**Authors:** Hongbiao Chen, Minyi Zhang, Lixian Su, He Cao, Xiaofeng Zhou, Zihao Gu, Huamin Liu, Fei Wu, Qiushuang Li, Juxian Xian, Qing Chen, Qihui Lin

**Affiliations:** ^1^Department of Epidemiology and Infectious Disease Control, Longhua Key Discipline of Public Health for the Prevention and Control of Infectious Diseases, Longhua Centre for Disease Control and Prevention, Shenzhen, China; ^2^Department of Epidemiology, School of Public Health, Southern Medical University, Guangzhou, China; ^3^Department of Child Healthcare, Futian Maternity and Child Healthcare Hospital, Shenzhen, China

**Keywords:** COVID-19, knowledge, attitude, practice, teacher

## Abstract

**Background:** Adequate understanding and precautionary behaviors are of vital importance to contain the spread of coronavirus disease 2019 (COVID-19). To date, the knowledge, attitudes, and practices (KAP) toward COVID-19 among different populations have been reported, whereas such information is unavailable in teachers. We aimed to investigate the KAP of teachers associated with COVID-19 during the global outbreak.

**Methods:** A large-scale population-based survey was conducted to gather information on COVID-19-related KAP among Chinese teachers using a self-administered questionnaire. We received 10,658 responses in April 2020, out of which 8,248 were enrolled in the final analysis. Participants responded to a self-administered questionnaire concerning demographic characteristics and KAP associated with COVID-19.

**Results:** This work included 4,252 (51.6%) teachers in kindergartens, 2,644 (32.1%) teachers in primary schools, and 1,352 (16.4%) teachers in secondary schools. The knowledge level (mean: 4.46 out of seven points) was relatively lower than the levels of attitudes (mean: 3.27 out of four points) and practices (mean: 4.29 out of five points) toward COVID-19. Knowledge scores significantly varied by the collected demographic variables except education worksite (*p* < 0.05), whereas practice scores significantly differed in age groups (*p* < 0.05), education level (*p* < 0.001), education worksite (*p* < 0.001), and years of teaching (*p* < 0.001). The multivariate logistic analysis indicated that poor knowledge related to COVID-19 was common among men, younger, and less-educated teachers. In contrast, female teachers and those with higher education levels tend to have good practices against COVID-19.

**Conclusion:** The present work suggested the knowledge gaps regarding COVID-19 were needed to be corrected immediately in teachers. Given the critical role of teachers in the education system, health authorities should take gender, age, and education level into account when developing suitable health interventions.

## Background

The coronavirus disease 2019 (COVID-19), caused by severe acute respiratory syndrome-coronavirus-2 (SARS-CoV-2), has been spreading worldwide since December 2019 (1). In the light of the World Health Organization (WHO), more than 119.2 million confirmed cases of COVID-19 were reported as of March 9, 2021, resulting in great threats and significant challenges to human health (2). The number of COVID-19 cases is still rising, and thereby the fierce fight against the spread of COVID-19 is continuing across the globe (2). Since January 2020, many Chinese regions were locked down, and the Chinese residents were required to stay at home to avoid contact with others. Apart from the closure of numerous shops, markets, and public spaces, schools and universities were also suspended opening during the rapid rise period of the COVID-19 outbreak. Other countries had also imposed similar measures to make every possible effort to curb further transmission of COVID-19 inside and outside the nations (3). Public health emergencies affect the education, safety, and mental health of adolescents around the world. During the Ebola epidemic in West Africa, for instance, many children dropped out of school due to education interruption, as school closure would be helpful to curb the spread of Ebola (4). The current COVID-19 pandemic also forced most governments to temporarily close educational institutions, influencing more than 91% of the student population worldwide (4).

It is commonly recognized that good knowledge, attitudes, and practices (KAP) among the public are of vital importance to guarantee the final success of the battle against pandemics (3, 5). For instance, KAP surveys of the SARS in 2003 implied that knowledge and attitudes regarding infectious diseases are related to the level of panic emotion among persons, resulting in complicated attempts to epidemic prevention and control (6, 7). In the case of novel influenza A (H1N1) in 2009, the knowledge was not optimistic among the general Chinese population as most of them confused the transmission route of H1N1, providing evidence to the government for preventive measures during the H1N1 pandemic (8). Jalloh et al. indicated that Ebola-related KAP directly informed the development of a national social mobilization strategy concerning Ebola virus disease (9). Overall, KAP assessments are widely applied to identify knowledge gaps and individual behaviors among different sociodemographic subgroups, providing effective interventions to improve public health (10, 11).

To date, a number of studies have been published focusing on COVID-19-related KAP within various populations, such as factory workers (12), general residents (5), income-poor households (13), medical-imaging professionals (14), university students (15), and healthcare workers (16). However, to the best of our knowledge, the COVID-19-related KAP has not been measured in the teacher population until now. As custodians of knowledge, the role of teachers is of paramount significance in the education system (17). A conclusion drawn from a review of seven published studies indicated that student mental health might be affected by their teachers (18). Another prior work reported that the attitudes of teachers and good practices had a crucial effect on their performance of the students (17). Hence, understanding the COVID-19-related KAP among the teachers is an urgent need at this critical moment.

In the present work, we conducted an online cross-sectional survey to determine the KAP related to COVID-19 among teachers engaged in kindergarten, primary, and secondary schools in China during the COVID-19 pandemic. Specifically, the purposes of our work included: (1) examining the levels of COVID-19-related KAP among the teacher population, (2) investigating the associations of KAP with demographic characteristics, and (3) identifying preferences of teachers regarding the sources of COVID-19 information in an attempt to facilitate health information acquisition.

## Methods

### Setting and Participants

In April 2020, we conducted an online cross-sectional investigation using a self-administered questionnaire in Shenzhen City *via* the most extensive online survey platform in China called Wen Juan Xing (Changsha Ranxing Information Technology Co., Ltd., Hunan, China). We invited 24,000 teachers engaged in kindergartens, primary schools, and secondary schools covering the whole of Longhua, an urban district in Shenzhen city, to determine the status of COVID-19-related KAP during the outbreak. Only the teachers who provided consent to be a part of the investigation were enrolled as participants. The initial data were collected from 10,658 individuals, with a response rate of 44.4%. The exclusion criteria included the following: (1) duplicate questionnaires submitted by participants with the same IP address (*n* = 2,387) and (2) respondents who took <100 s to complete the questionnaire (*n* = 23). A final total of 8,248 teachers without missing values were included in the present work (Supplementary Figure 1). The protocol for this work was approved by Longhua Center for Disease Control and Prevention (CDC), Shenzhen.

### Questionnaire

A self-administered questionnaire was developed for the present survey. Based on the literature review regarding the given topic, CDC researchers designed and prepared the first version of the questionnaire. Subsequently, we conducted a pretest using 50 teachers other than those who were included in this work. After that, the first version of the questionnaire was reviewed again and modified by CDC researchers and experts in epidemiology and infectious diseases. The questionnaire consisted of four parts covering baseline information and KAP related to the COVID-19 outbreak. Part I consisted of the general characteristics of the participants, including gender (male, female), age (≤30, 31–40, 41–50, and >50 years), education worksite (kindergarten, primary school, and secondary school), education levels (junior high school and below, high school, bachelor's degree, and master's degree or above), and years of teaching (≤2, 3–5, 6–10, and >10 years). Part II contained seven knowledge items to estimate the general understanding of clinical manifestation, transmission routes, and preventive measures associated with COVID-19 of the participants, including five multiple-choice items (K1–K5) and two single-choice items (K6–K7). For evaluation, a correct answer was given 1 point for each item, and otherwise, 0 point would be assigned according to the detailed scoring method, as described in Supplementary Table 1. The overall knowledge score ranged between 0 and 7, and the participants were separated into two groups based on their knowledge scores. The “good” knowledge group consisted of participants who scored more than the total sample median (median = 5), whereas the “poor” knowledge group consisted of participants who scored below the total sample median. Part III focused on the attitudes toward COVID-19 estimated by four items (A1–A4) with the maximum possible score of 4. Likewise, the participants were divided into two groups. Participants who obtained a score more than the sample median (median = 3) were regarded as indicating “good” attitudes, whereas those who scored below the median were regarded as indicating “poor” attitudes. Part IV included five items on practices (P1–P5) to estimate personal hygiene and behaviors during the pandemic. The maximum possible score was 5. After calculating the practice scores, the participants were also divided into two groups. The “good” practice group included participants who obtained a score of more than the sample median (median = 4), whereas a score below the median was assigned to a participant who was considered to denote “poor” practices. Similar to the work design of prior works (15), the knowledge section was developed based on the questions that have been used to assess COVID-19-related knowledge levels (5, 12), whereas the attitude and practice sections were self-developed by researchers based on the scenarios most likely encountered by the teacher population for the present work. A summary of KAP items is shown in Supplementary Table 1.

Moreover, three additional questions on acquiring information related to COVID-19 were included as follows: (1) Is it necessary to be very familiar with the prevention and control guidelines about infectious diseases on campus? (Very necessary, necessary, and not necessary); (2) How do you prefer to obtain COVID-19-related information? [Internet or social software (WeChat, Weibo, and Zhihu), school physician, lectures, videos, and newspaper/poster]; (3) What information do you prefer to obtain concerning COVID-19? (Knowledge about the novel coronavirus, personal preventive measures, hygiene and disinfection, and no knowledge required) (Supplementary Figure 2). All questions were close-ended and treated as categorical variables.

### Statistical Analyses

The categorical variables were presented as frequencies with percentages. The KAP scores of different persons, in the light of demographic variables, were described in the format mean ± standard deviation. They were compared with independent samples *t-*test or one-way analysis of variance test for normal distribution data, which was determined by using histograms and Q–Q plots. Binary logistic regression analyses were carried out to investigate factors associated with COVID-19-related KAP by calculating odds ratios (OR) and 95% confidence intervals (CI) after full adjustment for demographic variables, including gender, age, education level, education worksite, and years of teaching. Correlations between scores of KAP were also investigated, with Pearson correlation coefficients (*r*) being obtained. All analyses were generated in R statistical software v.4.0.3 (R Foundation for Statistical Computing, Vienna, Austria). We considered statistical tests with *p*-values <0.05 statistically significant. All *p*-values were two-sided.

## Results

### General Characteristics of the Subjects

Among all the 8,248 participants, most (89.2%) were women, and over half the participants (53.1%) were aged 30 years and younger; the group aged over 50 years accounted for only 3.7% of the participants. Among the participants, 40.7% attained a bachelor's degree, and 8.6% held a master's degree or above. Regarding educational institutions where the participants worked, 51.6% worked in kindergartens, whereas 32.1 and 16.4% worked in primary and secondary schools, respectively. Also, 27.8% of them have been engaged in educational duties for ≤2 years. Demographic characteristics are detailed in Table 1.

**Table 1:** General characteristics of participants, Shenzhen, China, April 2020.

**General characteristics**	**Total (** ***n*** **= 8,248)**		**95% CI**
	***N***	**%**	
Gender			
Male	892	10.8	10.2–11.5
Female	7,356	89.2	88.5–89.8
Age group (y)			
≤30	4,379	53.1	52.0–54.2
31–40	2,371	28.7	27.8–29.7
41–50	1,192	14.5	13.7–15.2
>50	306	3.7	3.3–4.1
Education level			
Junior high school or lower	1,458	17.7	16.9–18.5
High school	2,723	33.0	32.0–34.0
Bachelor's degree	3,358	40.7	39.7–41.8
Master's degree or above	709	8.6	8.0–9.2
Education worksite			
Kindergarten	4,252	51.6	50.5–52.6
Primary school	2,644	32.1	31.0–33.1
Secondary school	1,352	16.4	15.6–17.2
Years of teaching			
≤2	2,291	27.8	26.8–28.8
3–5	2,286	27.7	26.8–28.7
6–10	1,918	23.3	22.3–24.2
>10	1,753	21.3	20.4–22.2

### COVID-19 Knowledge

The accuracy rate of the seven items used to assess COVID-19-related knowledge ranged from 8.5 to 85.7%. For example, 79.4% of the participants correctly answered that the most common symptoms of COVID-19 included fever, fatigue, and dry cough. Most of the participants (85.6%) correctly responded that wearing a mask and exercising during the pandemic can prevent COVID-19 infection. In comparison, few participants (8.5%) correctly answered that vaccination was the safest, most effective, and economical measure against infectious diseases (Supplementary Table 1). The mean knowledge score was 4.46 ± 1.25 points, indicating a total correct rate of 63.7% (4.46/7*100) on the present knowledge survey (Table 2). The knowledge score significantly varied by gender (*p* < 0.001), age groups (*p* < 0.001), education levels (*p* < 0.001), and years of teaching (*p* < 0.001). Binary logistic analysis of COVID-19-related knowledge indicated that female teachers had a better knowledge of COVID-19 (OR 1.610, 95% CI 1.380–1.879). Individuals aged 31–40 years (OR 1.540, 95% CI 1.365–1.738) and 41–50 years (OR 1.823, 95% CI 1.543–2.154) had more knowledge of COVID-19 compared to those aged ≤ 30 years. Moreover, participants with an educational level of high school were more likely to have good COVID-19-related knowledge (OR 1.220, 95% CI 1.055–1.411) than those with a lower level of education. Teachers who held a bachelor's degree (OR 1.423, 95% CI 1.196–1.694) or a master's degree (OR 1.350, 95% CI 1.071–1.702) were also associated with good knowledge toward COVID-19 (Table 3).

**Table 2:** Knowledge, attitude, and practice scores of COVID-19 by demographic variables.

**Characteristics**	**Knowledge**			**Attitude**			**Practice**		
	**Score**	***t*/*F***	***P*-value**	**Score**	***t*/*F***	***P*-value**	**Score**	***t*/*F***	***P*-value**
Total	4.46 ± 1.25			3.27 ± 0.71			4.29 ± 0.79		
Gender		−4.375	<0.001		−1.103	0.270		−1.115	0.265
Male	4.28 ± 1.35			3.25 ± 0.71			4.26 ± 0.83		
Female	4.49 ± 1.23			3.27 ± 0.71			4.29 ± 0.79		
Age group (y)		47.688	<0.001		1.781	0.148		5.845	0.001
≤ 30	4.32 ± 1.25			3.28 ± 0.71			4.26 ± 0.81		
31–40	4.63 ± 1.21			3.24 ± 0.71			4.34 ± 0.77		
41–50	4.68 ± 1.22			3.29 ± 0.69			4.28 ± 0.77		
>50	4.40 ± 1.25			3.30 ± 0.68			4.25 ± 0.82		
Education level		8.745	<0.001		2.572	0.052		32.236	<0.001
Junior high school or lower	4.38 ± 1.31			3.23 ± 0.72			4.17 ± 0.82		
High school	4.42 ± 1.22			3.29 ± 0.69			4.23 ± 0.81		
Bachelor's degree	4.55 ± 1.22			3.27 ± 0.71			4.36 ± 0.77		
Master's degree or above	4.43 ± 1.35			3.27 ± 0.72			4.42 ± 0.74		
Education worksite		0.953	0.386		2.227	0.108		20.948	<0.001
Kindergarten	4.45 ± 1.23			3.27 ± 0.70			4.23 ± 0.80		
Primary school	4.49 ± 1.25			3.28 ± 0.71			4.34 ± 0.78		
Secondary school	4.48 ± 1.31			3.23 ± 0.73			4.36 ± 0.78		
Years of teaching		26.097	<0.001		0.303	0.823		6.863	<0.001
≤ 2	4.33 ± 1.31			3.26 ± 0.71			4.26 ± 0.83		
3–5	4.41 ± 1.24			3.27 ± 0.72			4.25 ± 0.80		
6–10	4.51 ± 1.21			3.26 ± 0.70			4.31 ± 0.78		
>10	4.66 ± 1.19			3.28 ± 0.70			4.35 ± 0.76		

**Table 3:** Factors associated with good knowledge, attitudes, and practices toward COVID-19 among the teacher population.

**Characteristics**	**Knowledge**			**Attitude**			**Practice**		
	**Good (%)**	**OR (95% CI)**	***P*-value**	**Good (%)**	**OR (95% CI)**	***P*-value**	**Good (%)**	**OR (95% CI)**	***P*-value**
Gender									
Male	410 (9.4)	1.000		778 (10.8)	1.000		738 (10.5)	1.000	
Female	3,949 (90.6)	1.610 (1.380–1.879)	<0.001	6,424 (89.2)	0.952 (0.758–1.194)	0.668	6,287 (89.5)	1.465 (1.190–1.805)	<0.001
Age group (y)									
≤ 30	2,099 (48.2)	1.000		3,838 (53.3)	1.000		3,696 (52.6)	1.000	
31–40	1,383 (31.7)	1.540 (1.365–1.738)	<0.001	2,043 (28.4)	0.907 (0.760–1.082)	0.277	2,058 (29.3)	1.175 (0.993–1.392)	0.061
41–50	724 (16.6)	1.823 (1.543–2.154)	<0.001	1,052 (14.6)	1.145 (0.892–1.469)	0.289	1,021 (14.5)	1.222 (0.972–1.537)	0.085
>50	153 (3.5)	1.294 (0.988–1.695)	0.061	269 (3.7)	1.142 (0.760–1.716)	0.523	250 (3.6)	0.974 (0.683–1.390)	0.886
Education level									
Junior high school or lower	744 (17.1)	1.000		1,254 (17.4)	1.000		1,181 (16.8)	1.000	**^#^**
High school	1,394 (32.0)	1.220 (1.055–1.411)	0.007	2,413 (33.5)	1.346 (1.086–1.668)	0.007	2,271 (32.3)	1.250 (1.034–1.511)	0.021
Bachelor's degree	1,856 (42.6)	1.423 (1.196–1.694)	<0.001	2,919 (40.5)	1.274 (0.986–1.646)	0.064	2,941 (41.9)	1.880 (1.482–2.384)	<0.001
Master's degree or above	365 (8.4)	1.350 (1.071–1.702)	0.011	616 (8.6)	1.350 (0.961–1.897)	0.083	632 (9.0)	2.397 (1.709–3.360)	<0.001
Education worksite									
Kindergarten	2,216 (50.8)	1.000		3,736 (51.9)	1.000		3,563 (50.7)	1.000	
Primary school	1,414 (32.4)	0.943 (0.830–1.072)	0.373	2,307 (32.0)	0.878 (0.724–1.065)	0.187	2,286 (32.5)	0.906 (0.755–1.086)	0.285
Secondary school	729 (16.7)	0.974 (0.822–1.154)	0.758	1,159 (16.1)	0.754 (0.588–0.966)	0.026	1,176 (16.7)	0.897 (0.700–1.148)	0.388
Years of teaching									
≤ 2	1,143 (26.2)	1.000		2,006 (27.9)	1.000		1,920 (27.3)	1.000	
3–5	1,150 (26.4)	0.956 (0.849–1.078)	0.462	1,981 (27.5)	0.893 (0.748–1.066)	0.211	1,924 (27.4)	1.032 (0.877–1.214)	0.708
6–10	1,021 (23.4)	0.937 (0.819–1.071)	0.340	1,683 (23.4)	1.002 (0.818–1.228)	0.984	1,653 (23.5)	1.106 (0.918–1.333)	0.289
>10	1,045 (24.0)	1.025 (0.861–1.219)	0.783	1,532 (21.3)	0.972 (0.751–1.256)	0.826	1,528 (21.8)	1.113 (0.871–1.421)	0.392

*OR, odds ratios; CI, confidence interval*.

# *P for trend <0.05*.

### COVID-19 Attitudes

The positive answer rate of the items on attitudes toward COVID-19 ranged between 45.7 and 98.2%. Over 80% of the participants believed they could actively respond to the COVID-19 pandemic despite feeling worried about it and could pay more attention to the prevention and control of infectious diseases in kindergartens or schools where they were. Nearly all the participants (98.2%) indicated that they would not decrease vigilance against COVID-19, even though the situation is witnessing positive changes. However, more than half of the participants (54.3%) felt pressure on working when the schools would reopen (Supplementary Table 1). The mean attitudes score was 3.27 ± 0.71 points out of 4 points, revealing a higher positive rate of 81.8% (3.27/4*100) on this series of attitude items (Table 2). At the same time, no statistical significance was observed between demographic variables and attitude scores. Results of the binary logistic analysis showed that participants teaching in secondary schools (OR 0.754, 95% CI 0.588–0.966) were significantly associated with poor attitudes toward COVID-19 vs. those in teaching in kindergartens. While having an education level of high school (OR 1.346, 95% CI 1.086–1.668) was a predictor of good attitudes compared with the education level of junior high school (Table 3), it should be noted that someone who did not attend high school but had obtained a kindergarten teacher certificate could be engaged as a kindergarten teacher in China.

### COVID-19 Practices

Among our participants, the most frequent practice was staying at home (95.3%) during the outbreak, followed by frequently washing hands with sanitizer (94.1%) and following the standards for wearing a mask (85.5%) during the period of COVID-19. Most participants (70.0%) have stored valuable articles such as sanitizer and drugs for further use against COVID-19 (Supplementary Table 1). The mean practices score was 4.29 ± 0.79 points on the 5-item scale, demonstrating that the best practice response rate was 85.8% (4.29/5*100) on this practice survey rather than investigation on COVID-19-related knowledge and attitudes (Table 2). Practice scores significantly differed across age groups (*p* = 0.001), education levels (*p* < 0.001), education worksite (*p* < 0.001), and years of teaching (*p* < 0.001). From the findings of the multivariate logistic analysis, female teachers (OR 1.465, 95% CI 1.190–1.805) were more likely to have good practices responding to COVID-19 than their male counterparts. Furthermore, individuals with higher education levels were significantly associated with good practices against COVID-19 (*p* for trend <0.05, junior high school or lower vs. master's degree or above, OR 2.397, 95% CI 1.709–3.360) (Table 3).

There was a significant positive correlation between knowledge–practices (*r* = 0.136, *p* < 0.001), knowledge–attitudes (*r* = 0.099, *p* < 0.001), and attitudes–practices (*r* = 0.074, *p* < 0.001) (Supplementary Table 2).

### Access to Obtain COVID-19-Related Information

The vast majority of the teachers (91.5%) agreed that it is quite necessary to understand campus prevention and control guidelines for infectious diseases. Regarding sources of acquiring information related to COVID-19, most participants preferred to attend lectures organized by the government (61.1%), followed by watching videos (55.9%) and internet or social software (WeChat, Weibo, and Zhihu, 42.4%). More than 70% of the participants were concerned about information in regard to the novel coronavirus, personal preventive measures, hygiene, and disinfection (Supplementary Figure 2).

## Discussion

To the best of our knowledge, the present work is the first to investigate COVID-19-related KAP among teachers based on a large-scale sample size during the global outbreak of COVID-19. Our findings indicated that the level of knowledge (mean score: 4.26 out of seven points) was relatively lower than the levels of attitudes (mean score: 3.27 out of four points) and practices (mean score: 4.29 out of five points) related to COVID-19.

At the time the survey was administered, most of the participants (79.4%) correctly answered the most common clinical symptoms of COVID-19, including fever, fatigue, and dry cough. In prior studies targeting this item, the correct answer rate was 85.1% in both Syrian and South Korean residents (3, 10) and 96.4% in the general Chinese population regarding the main symptoms of COVID-19 (5). We considered that the different levels of this item might be due to the different item-format. In most previous studies, the items were answered on a yes/true/agree, no/false/disagree basis, with an additional option of not sure/do not know (3, 5, 10, 12, 14). In comparison, the format of multiple-choice items in the present work might indicate the fundamental knowledge level. In this case, 85.7% of the participants answered the three appropriate transmission routes of COVID-19; but however, 27.6% of them wrongly replied that the COVID-19 could be disseminated through mosquito bites (Supplementary Table 1). This suggested that more education is needed to be provided by public health decision-makers and health-workers regarding the possible transmission routes of COVID-19.

In accordance with several surveys previously performed in China (5, 12), an overwhelming majority of our participants believed wearing a mask (99.7%) and doing exercise (96.6%) are adequate precautions to control and prevent the spread of COVID-19. However, wearing masks was recognized as an effective measure against COVID-19 by only 25.5% of the medical imaging professionals in India (14) and by 49.0% of income-poor households in the Philippines (13). These variations in knowledge levels might be attributed to the different precautions primarily implemented by countries. For the types of masks, most of the participants in the present work believed that N95 masks (90.8%) and medical–surgical masks (81.5%) are available to prevent COVID-19. However, we noted that some participants were confused about the appropriate types of the mask because they incorrectly answered dust masks (27.5%), activated carbon masks (13.6%), and cotton masks (2.8%) in this item (Supplementary Table 1). During the early stages of COVID-19, the National Health Commission of China issued protocols for rapid precautions to effectively contain the spread of the epidemic (19). Simultaneously, the masks were introduced as a valuable personal protective equipment to slow down the transmission of the COVID-19 (20). This knowledge gap in wearing an appropriate mask was an essential finding within the present work and needs to be corrected immediately.

We also asked the teachers a question about how to prevent and control the COVID-19 in classrooms. Most of the participants (80.1%) fully answered that they agreed to keep the classrooms well-ventilated, disinfect items every day, and observe the body temperature of each student. Under the national guidelines, it is vital to perform body temperature monitoring to contain cluster cases of COVID-19 in educational institutions (19). However, when it came to an item pertaining to precautions to help prevent the spread of infectious diseases, most of our participants (91.5%) failed to understand that vaccination was considered the safest, most effective, and most economical measure against COVID-19 (21, 22). Despite the fact that the development of COVID-19 vaccines had been progressing at an unprecedented rate (21), we presumed the insufficient knowledge about vaccinations against COVID-19 might be due to the lack of available vaccines as of April 2020. The National Health Commission of China officially included COVID-19 into the management of class B notifiable infectious diseases on January 20, 2020 and followed class A notifiable infectious disease prevention and control steps (23, 24). Nevertheless, only 61.7% of our participants correctly responded that COVID-19 is a notifiable infectious disease in China (Supplementary Table 1). Hence, public health messaging of COVID-19 needs to be improved to deal with these knowledge gaps in the teacher population.

Female teachers showed a higher level of knowledge related to COVID-19 than men (*P* < 0.001). In line with previous work, women were more likely than men to listen to medical experts and extend their knowledge from the experiences of other countries and social media in response to COVID-19 (25). Compared with participants aged <30 years, those aged from 31 to 50 years had a significantly higher level of COVID-19-related knowledge (both *p* < 0.001). Also, the expected findings of better understanding of COVID-19 were observed in participants with a degree of high school (*P* = 0.007), bachelor's degree (*P* < 0.001), a master's degree, or above (*P* = 0.011) (Table 3). These highlighted that efforts should be directed toward increasing the knowledge of COVID-19 among men, younger, and less-educated teachers.

In agreement with attitude levels of COVID-19 documented among Chinese undergraduate students and the general Iranian population (15, 26), most of our work participants displayed positive attitudes toward COVID-19, illustrating that health education was effectively delivered throughout kindergartens and schools in Shenzhen. Interestingly, over half of the participants expressed their feeling of pressure to teach when schools reopen in the postpandemic era of COVID-19 (Supplementary Table 1). Teachers from secondary schools showed a poor attitude about COVID-19 when compared with teachers working in kindergartens. The different educational situations could explain these findings among schools in China. In contrast to kindergartens, there are a larger number of students in the secondary schools, leading to much more complexity in management and teaching settings. Additional studies are needed to implement mental health exams among teachers in educational institutions. Moreover, the educational department should pay more attention to strengthening mental health care for the teachers and providing a more friendly educational environment.

The findings of COVID-19-related practices indicated that most participants (95.3%) took precautions to prevent COVID-19 infection by staying at home as much as possible during the outbreak, which was consistent with previous reports on COVID-19-related KAP in China (5, 12). This result could be primarily attributed to the strict prevention and control measures implemented by the Chinese governments in response to the severe situation, such as strict home quarantine, suspending public transportation, banning travel, and public gatherings, among other precautionary measures (3). As suggested by results of prior studies regarding gender patterns of risk-taking behaviors (27, 28), men have a greater likelihood of engaging in risk-taking behaviors. Meanwhile, a previous survey queried nearly 800 U.S. residents, and it showed that women had more tendency than men to embrace behaviors to prevent the spread of COVID-19, such as maintaining social distance, staying at home, and frequency of handwashing (29). The findings of our work were in accordance with these prior results that female teachers were significantly more likely to engage in good practices to help prevent the spread of COVID-19. As expected, our findings of a statistical trend between education levels and COVID-19-related practices were generally consistent with previous reports (5, 12), indicating that higher education levels were found to be significantly correlated with a higher likelihood of engaging in good practices toward COVID-19 (*p* for trend <0.05). These results further suggested that health education intervention ought to be improved targeting specific demographic groups, especially for male teachers and those with lower education levels. Furthermore, our findings provided evidence of the significant positive correlations between knowledge–attitudes, knowledge–practices, and attitude—practices among the participants. These indicated higher COVID-19-related knowledge and attitudes scores associated with having good practices against COVID-19 in the present survey (Supplementary Table 2). These findings were in line with previous studies (5, 10), bringing to light that preventive behaviors could be heightened by improving their knowledge and attitudes toward COVID-19.

With respect to the source of COVID-19 information, most of our respondents indicated they would like to receive information through lectures, videos, and internet or mobile social software such as WeChat and Weibo. This result conformed to a prior KAP survey that targeted factory workers in China, indicating that new media, especially mobile social software, plays an essential role in disseminating public health messages associated with COVID-19 (12). Nonetheless, a cross-sectional survey among income-poor households in the Philippines showed that most participants preferred to acquire COVID-19 messages through traditional media sources, such as television and radio, and the limited internet connectivity in multiple areas in the Philippines supported this result (13, 30). Taken together, the use of public health communication strategies should be adapted to local conditions to provide the public with knowledge and guide them to take adequate measures when public emergencies arise.

The strength of this survey, firstly, lies in the large-scale population recruited during a critical period. Secondly, the format of multiple-choice items could reflect the actual knowledge level of the teachers related to COVID-19. Thirdly, the questionnaire was developed based on previous studies and through content validation. However, this work has several potential limitations that should be acknowledged. The targeted population only consisted of teachers from a single city that was not representative of all teachers in China. Given this critical moment, the information collected in this work might be subject to recall bias due to the use of self-reported questionnaires.

## Conclusion

In summary, this large population-based work aimed at evaluating KAP toward COVID-19 among the teachers from kindergartens, primary schools, and secondary schools in Shenzhen, China. These results showed lower levels of COVID-19-related KAP among men, younger, and less-educated teachers. Given the critical role of teachers in the education system, this work is expected to help health and education authorities formulate suitable interventions to address the knowledge gaps of teachers.

## Data Availability Statement

The raw data supporting the conclusions of this article will be made available by the authors, without undue reservation.

## Ethics Statement

The studies involving human participants were reviewed and approved by Longhua District Centre for Disease Control and Prevention. Written informed consent was not provided because we had obtain oral informed consent.

## Author Contributions

HCh, MZ, LS, and QLin contributed to design the framework of this work. HCa, XZ, and ZG contributed to the formal analysis. Data collection was performed by FW, QLi, and JX. The original draft of the manuscript was written by MZ and HCh. HL, QC, and QLin contributed to the manuscript review and editing. All authors have read and agreed to the published version of the manuscript.

## Conflict of Interest

The authors declare that the research was conducted in the absence of any commercial or financial relationships that could be construed as a potential conflict of interest.

## Publisher's Note

All claims expressed in this article are solely those of the authors and do not necessarily represent those of their affiliated organizations, or those of the publisher, the editors and the reviewers. Any product that may be evaluated in this article, or claim that may be made by its manufacturer, is not guaranteed or endorsed by the publisher.

## Abbreviations

KAP: knowledge, attitudes, and practices
COVID-19: Coronavirus disease 2019
SARS-CoV-2: severe acute respiratory syndrome-coronavirus-2
WHO: World Health Organization
SARS: severe acute respiratory syndrome
H1N1: the novel influenza A
CDC: Center for Disease Control and Prevention
OR: odds ratio
CI: confidence interval.
