# Supplementary material for: Knowledge, Attitudes, and Practices Toward COVID-19 Among Chinese Teachers, Shenzhen: An Online Cross-sectional Study During the Global Outbreak of COVID-19
Source: Front Public Health. 2021 Aug 20;9:706830. doi: 10.3389/fpubh.2021.706830 (PMC8417721; doi:10.3389/fpubh.2021.706830)
Supplement: Supplementary file 1 [file Data_Sheet_1.docx]

**Supplementary Material ID: 706830 KAP toward COVID-19**


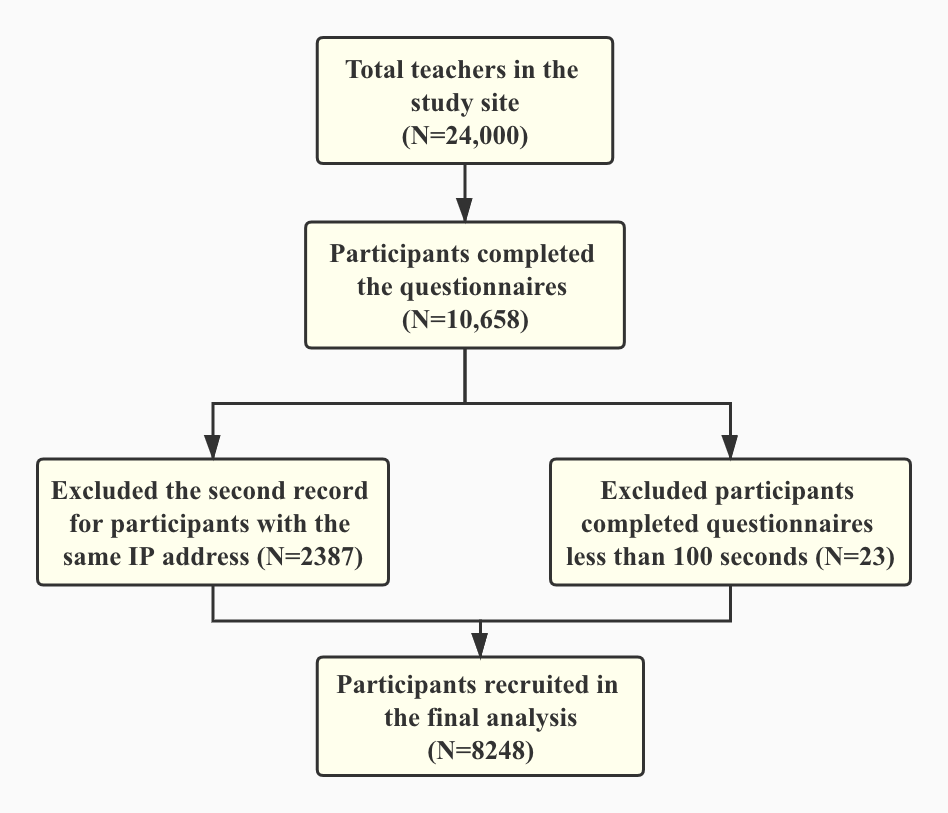


**Figure S1** Flowchart of participants enrollment

**Table 1** Summary of items for knowledge, attitudes and practices toward COVID-19 among the Chinese teacher in Shenzhen, April 2020

| Items | Options | Number (%) | 95% CI |
| --- | --- | --- | --- |
| **Knowledge** (Correct rate, % of the total sample) |  |  |  |
| K1. The most common symptoms of COVID-19 include (multiple-choice item) ^a^: (79.4%) | **Fever** | 8156 (98.9%) | 98.6-99.1 |
|  | **Fatigue** | 7548 (91.5%) | 90.9-92.1 |
|  | Rash | 484 (5.9%) | 5.4-6.4 |
|  | Diarrhea | 2301 (27.9%) | 26.9-28.9 |
|  | **Dry cough** | 7083 (85.9%) | 85.1-86.6 |
| K2. COVID-19 can be transmitted by (multiple-choice item) ^b^: (85.7%) | **Coughing and sneezing** | 8231 (99.8%) | 99.7-99.9 |
|  | Mosquito bites | 2280 (27.6%) | 26.7-28.6 |
|  | **Hugs and kisses** | 7449 (90.3) | 89.7-90.9 |
|  | **Touching wounds or eyes after toughing contaminated items** | 7694 (93.3) | 92.7-93.8 |
| K3. COVID-19 can be effectually prevented by (multiple-choice item) ^c^: (85.6%) | **Wearing a mask** | 8226 (99.7%) | 99.6-99.8 |
|  | Taking antibiotics | 548 (6.6%) | 6.1-7.2 |
|  | Closing windows to prevent virus to entering | 510 (6.2%) | 5.7-6.7 |
|  | **Exercise** | 7969 (96.6%) | 96.2-97.0 |
| K4. In the classrooms, COVID-19 can be prevented by (multiple-choice item) ^c^: (80.1%) | Closing doors and windows | 156 (1.9%) | 1.6-2.2 |
|  | **Keeping ventilation and disinfecting items every day** | 8209 (99.5) | 99.4-99.7 |
|  | Cleaning contaminated items with ordinary detergent | 1403 (17.0%) | 16.2-17.8 |
|  | **Body temperature monitoring** | 8082 (98.0%) | 97.7-98.3 |
| K5. Suitable masks for preventing COVID-19 include (multiple-choice item) ^c^: (45.5%) | **N95 mask** | 7493 (90.8%) | 90.2-91.5 |
|  | Dusk mask | 2271 (27.5%) | 26.6-28.5 |
|  | **Medical-surgical mask** | 6719 (81.5%) | 80.6-82.3 |
|  | Activated carbon mask | 1122 (13.6%) | 12.9-14.4 |
|  | Cotton mask | 228 (2.8%) | 2.4-3.1 |
| K6. The safest, most effective and most economical measure to prevent infectious diseases is (single-choice item): | Early detection, diagnosis, and treatment | 7525 (91.2%) | 90.6-91.8 |
|  | To see a doctor | 26 (0.3%) | 0.2-0.5 |
|  | **Vaccinations** | 697 (8.5%) | 7.9-9.1 |
| K7. The COVID-19 is a notifiable infectious disease in China (single-choice item). | **Yes** | 5087 (61.7%) | 60.6-62.7 |
|  | No | 3161 (38.3%) | 37.3-39.4 |
| **Attitudes** |  |  |  |
| A1. I would aggressively respond to the COVID-19. | **Yes** | 7170 (86.9%) | 86.2-87.7 |
|  | No | 1078 (13.1%) | 12.3-13.8 |
| A2. I pay more attention to the prevention and control of infectious diseases in kindergartens or schools during the pandemic than ever. | **Yes** | 7224 (87.6%) | 86.9-88.3 |
|  | No | 1024 (12.4%) | 11.7-13.1 |
| A3. I could not decrease vigilance against COVID-19 even if the situation is witnessing positive changes. | **Yes** | 8103 (98.2%) | 97.9-98.5 |
|  | No | 145 (1.8%) | 1.5-2.1 |
| A4. I feel pressure on working when reopening schools during the post-pandemic era of COVID-19. | Yes | 4475 (54.3%) | 53.2-55.3 |
|  | **No** | 3773 (45.7%) | 44.7-46.8 |
| **Practices** |  |  |  |
| P1. I frequently wash my hands with sanitizer during the pandemic. | **Yes** | 7760 (94.1%) | 93.6-94.6 |
|  | No | 488 (5.9%) | 5.4-6.4 |
| P2. I stay at home as much as possible during the pandemic. | **Yes** | 7859 (95.3%) | 94.8-95.7 |
|  | No | 389 (4.7%) | 4.3-5.2 |
| P3. I store some useful items, such as sanitizer and drugs, for further use during the pandemic. | **Yes** | 5770 (70.0) | 69.0-70.9 |
|  | No | 2478 (30.0%) | 29.1-31.0 |
| P4. I follow the standard for wearing a mask during the pandemic. | **Yes** | 7049 (85.5%) | 84.7-86.2 |
|  | No | 1199 (14.5%) | 13.8-15.3 |
| P5. I wear a N95 or medical surgical mask during the pandemic. | **Yes** | 6931 (84.0%) | 83.2-84.8 |
|  | No | 1317 (16.0%) | 15.2-16.8 |

CI, confidence interval.

Bold font represents the correct, positive or expected answers for these items.

^a^ Participants would get 1 point if they responded that the most common symptoms of COVID-19 included fever, fatigue, and dry cough, regardless of the other two options.

^b^ Participants would get 1 point if they responded that COVID-19 can be transmitted by coughing and sneezing, hugs and kisses, and touching wounds or eyes after toughing contaminated items, regardless of the other one option.

^c^ Participants would get 1 point only if they responded to all of the correct answers (in bold font), and otherwise, 0 point would be assigned for any incorrect or incomplete answers.


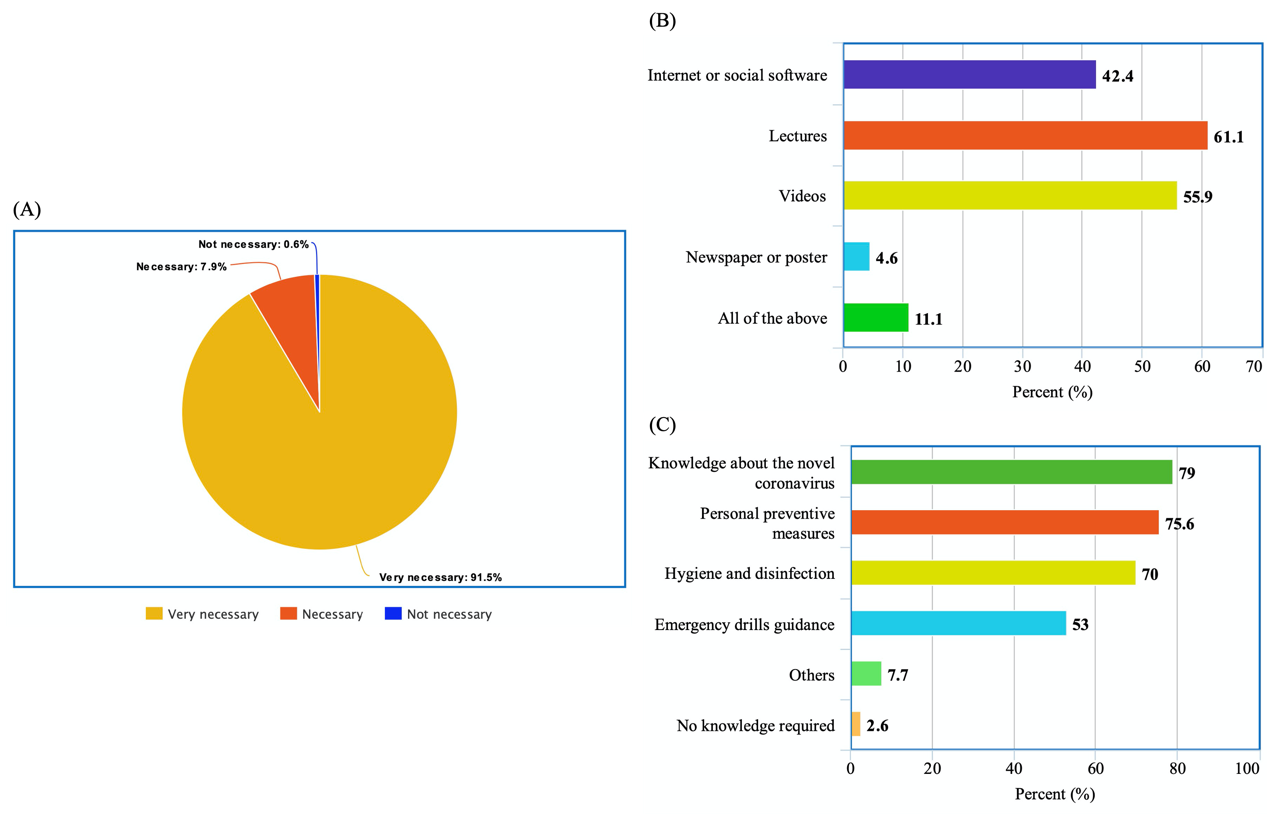


**Figure 2** Additional questions on acquiring information related to COVID-19

1. Participants were asked “Is it necessary to be very familiar with the prevention and control guidelines about infectious diseases on campus?”
2. Participants were asked “How do you prefer to obtain COVID-19-related information? (multiple-choice item)”
3. Participants were asked “What information do you prefer to obtain concerning COVID-19? (multiple-choice item)”

**Table 2** Correlations between scores of knowledge, attitudes, and practices towards COVID-19

| Variable | Knowledge | Attitudes | Practices |
| --- | --- | --- | --- |
| Knowledge | 1 | - | - |
| Attitudes | 0.099 ^a^ | 1 | - |
| Practices | 0.136 ^a^ | 0.074 ^a^ | 1 |

^a^ Correlation is significant at the 0.01 level (2-tailed).
